# Lithium compromises the bioenergetic reserve of cardiomyoblasts mitochondria

**DOI:** 10.1007/s10863-024-10050-x

**Published:** 2025-01-24

**Authors:** Marian Grman, Maria Balazova, Anton Horvath, Katarina Polcicova, Katarina Ondacova, Jakub Stepanovsky, Zuzana Sevcikova Tomaskova

**Affiliations:** 1 Institute of Clinical and Translational Research, Biomedical Research Center of the Slovak Academy of Sciences, Dubravska cesta 9, Bratislava, 845 05 Slovakia; 2 Institute of Animal Biochemistry and Genetics, Centre of Biosciences of the Slovak Academy of Sciences, Dubravska cesta 9, Bratislava, 84005 Slovakia; 3 Faculty of Natural Sciences, Comenius University, Mlynska dolina, Ilkovicova 6, Bratislava, 842 15 Slovakia; 4 Institute of Virology, Biomedical Research Center of the Slovak Academy of Sciences, Dubravska cesta 9, Bratislava, 845 05 Slovakia; 5 Institute of Molecular Physiology and Genetics, Centre of Biosciences of the Slovak Academy of Sciences, Dubravska cesta 9, Bratislava, 840 05 Slovakia; 6 Faculty of Chemical and Food Technology, Slovak Technical University, Radlinskeho 9, Bratislava, 812 37 Slovakia; 7 Present Address: Institute of Clinical and Translational Research, Biomedical Research Center, Slovak Academy of Sciences, Dubravska cesta 9, Bratislava, 845 05 Slovakia

**Keywords:** Cellular respiration, Mitochondrial network, Cardiomyoblast, Lithium, Bipolar disorder

## Abstract

Lithium is used in the long-term treatment of bipolar disorder, exhibiting a beneficial effect on the neuronal cells. The concentration of lithium in the blood serum can vary and can easily approach a level that is related to cardiotoxic adverse effects. This is due to its narrow therapeutic index. In this study, we investigated the effect of higher than therapeutic dose of lithium. Rat cardiomyoblast cells were treated with 2 mM LiCl for 48 h, after which the mitochondrial parameters of the cells were analyzed. Lithium exposure reduced maximal respiratory capacity by diminishing reserve respiratory capacity (RRC), linked to a decrease in complex I (NADH dehydrogenase) activity and elevated superoxide radical levels. In addition, lithium treatment altered the composition of cellular membranes, including mitochondrial cardiolipin, a lipid essential for mitochondrial function. These findings suggest that impaired complex I activity, oxidative stress, and cardiolipin depletion collectively impair the ability of cells to meet high energy demands.

**Supplementary Information:**

The online version contains supplementary material available at 10.1007/s10863-024-10050-x.

## Introduction

Lithium has been used for the treatment of bipolar disorder (BD) for several decades. The beneficial effect of lithium was first identified in 1949 by John Cade (Cade 1949). The National Institute of Mental Health defines BD as a mental illness characterized by mood changes between episodes of high activity (mania or hypomania) and depressive episodes. Lithium remains the preferred treatment for mood stabilization. The key advantages of lithium are that it can be used as monotherapy for a longer period of time than other psychotropic drugs and that it has a significant effect in preventing suicidal behavior (Baldessarini et al. 2007; Tondo et al. 2019). The practical application was tied to the advent of methodologies for monitoring lithium level in the blood serum (Tondo et al. 2019). It was necessary for the safety of the treatment, given that the toxic level of lithium is very close to the therapeutic level, i.e. it has a narrow therapeutic index. The therapeutic range of lithium in blood serum is 0.5–1.2 mEq/L. However, due to the fluctuations in concentration shortly after dose administration or as a result of co-treatment that can affect the excretion of lithium from the body, its monitoring is essential. The administration of lithium has been linked to the emergence of adverse effects on the cardiovascular system. The case studies of patients exhibiting lithium-induced cardiotoxicity predominantly exhibit a common feature – namely an elevated concentration of lithium in blood serum (> 1.5 mEq/L) (Acharya et al. 2020; Kalpakos et al. 2022; Menegueti et al. 2012; Snipes et al. 2021). The spectrum of cardiovascular adverse effects encompasses a range of conditions that result in alterations of cardiac rhythm and other electrocardiogram parameters, including prolongation of PR or QT interval, Brugada pattern, various conductive blocks and changes in conduction velocity or increased incidence of hypertension (Klumpers et al. 2004; Mehta and Vannozzi 2017).

Salimi et al. demonstrated that acute exposure to the lithium (5–180 min) resulted in an elevation of reactive oxygen species (ROS) level in isolated cardiac mitochondria. In this setting, the metabolism of 3-(4,5-dimethylthiazol-2-yl)-2,5-diphenyltetrazolium bromide (MTT compound) was found to be reduced even in the presence of 1 mM lithium (Salimi et al. 2017), which is a concentration well within the therapeutic range. This prompted us to undertake a more detailed examination of mitochondrial bioenergetics in intact cardiomyoblast cells exposed to 2 mM lithium over an extended period (48 h). We used cardiomyoblast cell line, which can be cultivated for a long period of time, unlike the isolated primary cardiomyocytes. Cardiomyoblasts are a commonly utilized alternative to primary cardiomyocytes (Branco et al. 2015; Hescheler et al. 1991).

## Materials and methods

### Cell culture

The rat cardiomyoblast cells (h9c2 cell line, www.atcc.org, RRID: CVCL_0286) were cultured in high glucose Dulbecco's modified Eagle medium (Biosera) supplemented with 10% fetal bovine serum and penicillin/streptomycin (10% DMEM). The cells were counted on CASY cell counter and analyzer (Omni Life Science) and seeded at a density of 2-3x10^4^/cm^2^. After 24 h, the medium was changed for either control (10% DMEM) or lithium-containing medium (2 mM LiCl in 10% DMEM). The analyses were performed 48 h after the change of medium, 2 mM LiCl was present also in the solutions during the measurement of the lithium-treated groups. 2 mM LiCl corresponds to 2 mEq/L. LiCl was purchased from Sigma Aldrich (L4408).

### Cell counting

The cells were seeded at density of 2x10^4^ per cm^2^ in 12-well plates. After 48 h treatment with lithium, the cells were thoroughly washed 3x with phosphate buffered saline (PBS) and then exposed to 100 µL of trypsin solution (Sigma Aldrich, T3924) for 4 minutes at 37 °C. The detachment of the cells was checked by visual inspection under optical microscope. 200 µL of DMEM or lithium-containing DMEM was added and the cells were triturated to obtain a homogenous suspension of cells. The cells were then counted on CASY cell counter and analyzer (Omni Life Science). Each value is obtained as average of a triplicate measurement. The data were normalized according to (Valcu and Valcu 2011). All data were divided by the mean of control group prior to the statistical analysis.

### Cell proliferation assay

The cells were seeded in 96-well plates. After 48 h incubation in 2 mM LiCl in 10% DMEM or control medium, the CellTiter 96 AQ_ueous_ One Solution Cell Proliferation Assay (Promega) was performed according to the provided general protocol. The cells were incubated for 2 h at 37 °C in the dark and then the absorbance was measured at 490 nm. Each biological replicate was measured in 5 technical replicates. The resulting absorbance was normalized to µg of proteins. The amount of proteins was determined for each biological replicate separately using QuantiPro BCA Assay Kit (Sigma Aldrich) according to the manufacturer’s protocol. RIPA buffer (Serva) was used for the lysis of the cells.

### Propidium iodide staining

The staining with propidium iodide (1 µg/mL) was used in parallel to Hoechst (1 µg/mL) staining in order to determine the ratio of dead/live cells. PI does not enter into live cells. The cells were analyzed under Leica SP8 STED 3X confocal microscope with water immersion 20x objective, zoom 0.75, scanning speed 400 Hz and sequential scanning was used for separate imaging of these two dyes. Hoechst was excited at 405 nm and its emission was detected in range 410–535 nm. Propidium iodide was detected in a separate sequence, with excitation at 534 nm and emission in range 553–784 nm. The images covered an area of 775 μm x 775 μm. The number of nuclei in each image was detected using *ComDet v.0.5.5.* plugin in ImageJ (https://github.com/UU-cellbiology/ComDet).

### Oxygen consumption

After 48 h incubation in lithium, the cells were harvested using trypsin. The rate of oxygen consumption of intact cells (> 3.10^6^ cells per measurement) was measured using Mitocell MS200A (Strathkelvin Instruments). The rate of oxygen consumption was measured in a closed-cell arrangement in culture medium according to a standard protocol for intact cell respiration measurement (Hill et al. 2012). After basal respiration recording, 2 µM oligomycin A (Enzo) was added for the estimation of the proton leak rate. Maximal respiration capacity (maxRC) was induced by sequential addition of up to 15 µM FCCP (carbonyl cyanide 4-(trifluoromethoxy) phenylhydrazone; Sigma, step 2.5 µM) to achieve the plateau of maximal oxygen consumption. It was followed by addition of 2 µM rotenone (Sigma) and 2.5 µM antimycin A (Sigma) to estimate the rate of non-mitochondrial oxygen consumption. The rate of oxygen consumption (pmol O_2_ min^− 1^) was normalized to 10^6^ cells.

### Mitochondrial morphology

The cells were seeded on the 35 mm Glass bottom MatTek Petri dishes. After 48 h incubation in 2 mM LiCl in 10% DMEM, the cells were washed with PBS and loaded for 30 min at 37 °C in dark with 100 nM Mitotracker Deep Red (Invitrogen) in phenol red-free high-glucose Dulbecco modified Eagle medium (phenol red-free DMEM; Biosera). Confocal images were acquired using Leica SP8 STED 3X confocal microscope with oil immersion 63x objective, zoom 2.5, optical Sect. 0.89 μm and scanning speed 400 Hz. The excitation wavelength was set to 641 nm and emission was detected in range 651-784nm. The mitochondrial network was analyzed in ImageJ using *Mitochondria Analyzer* plugin (Chaudhry et al. 2020).

### Qualitative comparison of mitochondrial membrane potential

The cells were seeded on the 35 mm Glass bottom MatTek Petri dishes and after 48 h incubation with 2 mM LiCl in 10% DMEM were washed with PBS and loaded with 5 µg/mL JC-1 (Enzo) for 15 min at 37 °C in the dark, then washed twice with PBS and visualized in phenol red-free DMEM under Leica SP8 STED 3X confocal microscope with oil immersion 63x objective, zoom 1, optical Sect. 0.89 μm and scanning speed 400 Hz. Excitation of 490 nm was used and emission was detected on two detectors: green fluorescence in range 515–550 nm and red fluorescence in range 575–625 nm, according to Leica settings for JC-1 fluorophore. The fluorescence intensity was quantified in ImageJ as integrated density corrected to background fluorescence: *integrated density – (cell area x mean background fluorescence)*. JC-1 signal is expressed as ratio of red to green fluorescence. The JC-1 signal was detected also in presence of FCCP at three different concentrations: 5, 10 and 15 µM.

### Superoxide radical detection

The production of superoxide radicals by mitochondria in intact h9c2 cells was detected using 5 µM MitoSOX red (Invitrogen) for 10 min at 37 °C in dark, then washed twice with PBS and visualized in phenol red-free DMEM under confocal microscope Leica SP8 STED 3X confocal microscope with oil immersion 63x objective, zoom 1, optical Sect. 0.89 μm and scanning speed 400 Hz. Excitation wavelength was 405 nm; emission was detected in range 570–640 nm. The fluorescence intensity was quantified in ImageJ as integrated density corrected to background fluorescence: *integrated density – (cell area x mean background fluorescence)*.

### Activity of electron transport chain complexes

The activity of complexes I - IV was measured on lysates of mitochondria isolated from h9c2 cells, seeded and incubated as described for oxygen consumption measurements. The cells were harvested using trypsin, centrifuged at 500x*g* for 5 min, resuspended in 250 mM sucrose in 5 mM HEPES, Tris, pH 7.3, homogenized with Potter-Elvehjem homogenizer (60 strokes on ice). Unbroken cells were removed by centrifugation at 150x*g* for 5 min and the supernatant was centrifuged at 15,000x*g* for 15 min (4 °C). The pellet was resuspended in PBS or 2 mM LiCl-containing PBS, centrifuged again at 15,000x*g* for 15 min (4 °C). The mitochondrial pellets were lysed and the measurement of individual activities of complex I-IV were performed as described in (Horonyova et al. 2024). 2 mM LiCl was present in all solutions during the preparation and measurement of lithium-treated group.

### Western blot analysis

For western blot analysis, h9c2 cells were lysed in RIPA buffer (50 mM Tris /HCl, pH 7.4, 150 mM NaCl, 1 mM EDTA, 0.5% sodium deoxycholate, 1% Triton X-100, 0.1% SDS, protease inhibitors (Roche)) and the protein concentration was determined using Pierce BCA Protein Assay Kit (ThermoScientific). 80–100 µg of proteins were subjected to SDS-PAGE on a 10% gel or 12%gel (for complex IV detection) and transferred to a PVDF membrane (Amersham). The membrane was blocked overnight in blocking solution (5% BSA, 0.1% Tween 20 in Tris-buffered saline, pH 7.6). The membrane was incubated with primary anti-NADH dehydrogenase, subunit 2 (complex I; 1:500; Proteintech 19704-1-AP); anti-SDHA antibody (complex II; 1:20,000; Proteintech 66588-1-Ig); anti-ubiquinol-cytochrome c reductase core protein I (complex III; 1:4,000; Proteintech 21705-1-AP) and anti-cytochrome c oxidase subunit IV antibody (complex IV; 1:1000; Cell Signaling #11967), followed by HRP-conjugated goat anti-mouse secondary antibody (A0168, Merck; 1:10,000) or HRP-conjugated goat anti-rabbit secondary antibody (1:5,000; DAKO P0448). As a loading control, tubulin was detected using rabbit anti-tubulin antibody (1:10,000; Abcam ab4074) and IRDye^®^ 800RD donkey anti-rabbit secondary antibody (1:10,000; Li-Cor). The signal was detected using Chemi-doc (Biorad). The analysis was performed for 3 biological replicates. The blot images were quantified using ImageJ software.

### Phospholipid quantification

Lipids were extracted from the h9c2 cell homogenate in chloroform: methanol: HCl solution (with ratios 60:30:0.26), as is described in details in (Horonyova et al. 2024). Shortly, the extracted phospholipids were separated by 2D thin-layer chromatography (TLC) on Silica gel 60 plates (Merck) and visualized by staining with iodine vapor and the phosphate. The chromatograms were quantified as was described in our previous studies (Horonyova et al. 2024; Kubalova et al. 2019).

### Statistical analysis

All data were subjected to d’Agostino & Pearson Normality test and, subsequently, appropriate test was used for the determination of statistical significance - t-test for Normally distributed data, Mann-Whitney test for non-Normally distributed data. The probability value *P* < 0.05 was considered statistically significant. Normally distributed data are represented by mean and standard error of the mean (SEM) of N values. Non-Normally distributed data are represented by median and interquartile range (IQR) of N values. GraphPad Prism 10 software (GraphPad Software, San Diego, CA) was used for statistical analysis and graph preparation.

## Results

**Fig. 1:**
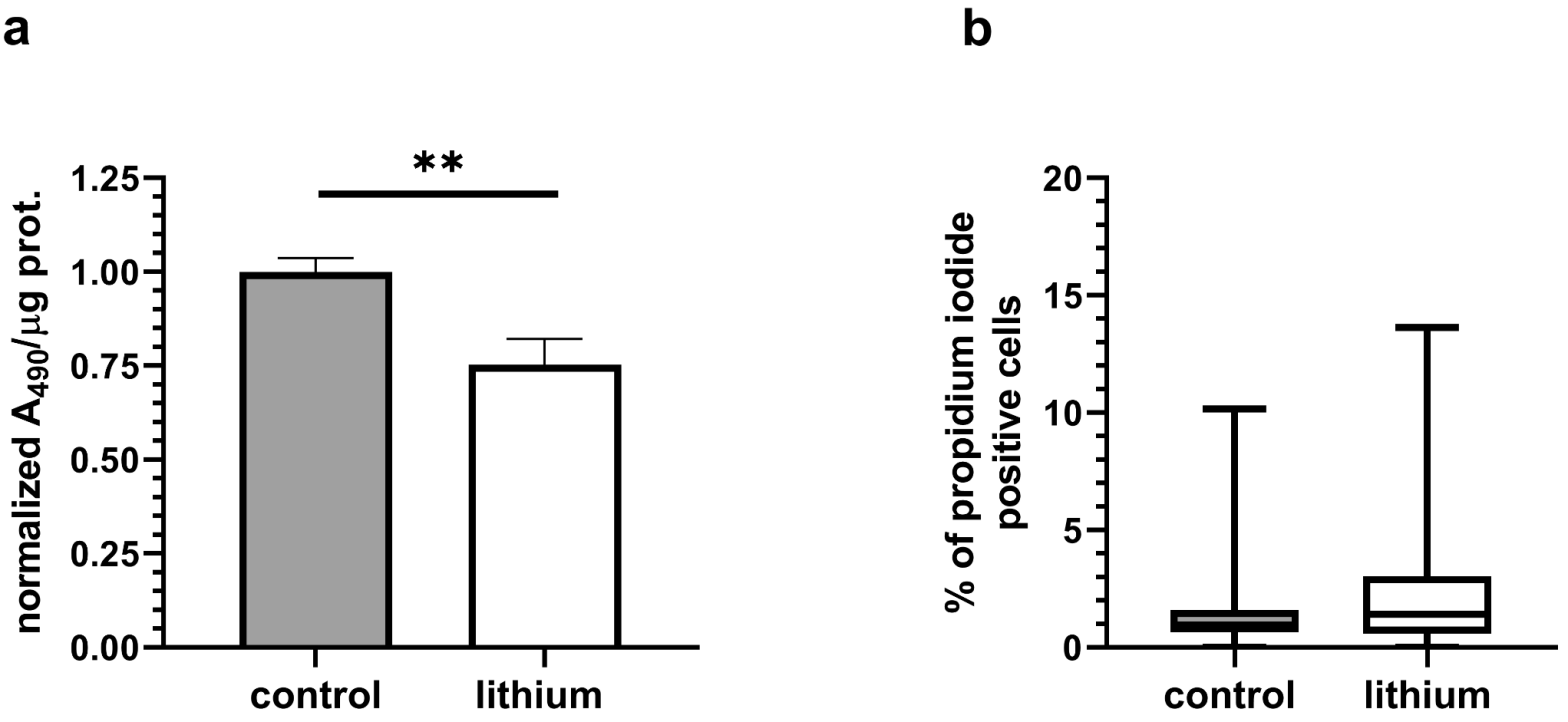
Cell proliferation assay (**a**) and propidium iodide staining (**b**). The absorbance of the water-soluble formazan produced from MTS compound at 490 nm was reduced in presence of lithium (t-test, *P* = 0.007; *N* = 8). The propidium iodide staining did not reveal significantly higher proportion of dead or defective cells (Mann-Whitney, *P* = 0.1186; N of analyzed images in control: N_control_=32 (total number of analyzed cells 9,048); in lithium group N_lithium_=41 (total number of analyzed cells 12,547))

In order to ascertain the effect of lithium on the cardiomyoblast cell line, we initially tested the viability of the cells exposed to 2 mM concentration of LiCl. The number of cells was found to be reduced in the presence of 2 mM lithium, representing only 80 ± 3% of the control cell count (*N* = 17, Online Resource, SI1). The cell proliferation assay based on the metabolism of MTS to water-soluble formazan was used to assess the impact of lithium on the cell viability. We observed a decrease in the absorbance of the formazan (Fig. 1a). The ratio of cells with defective membranes, which are sensitive to propidium iodide staining, was low in both groups (Fig. 1b; Online Resource, SI2), indicating that the lower cell count is due to slower proliferation rather than induction of cell death.

**Fig. 2:**
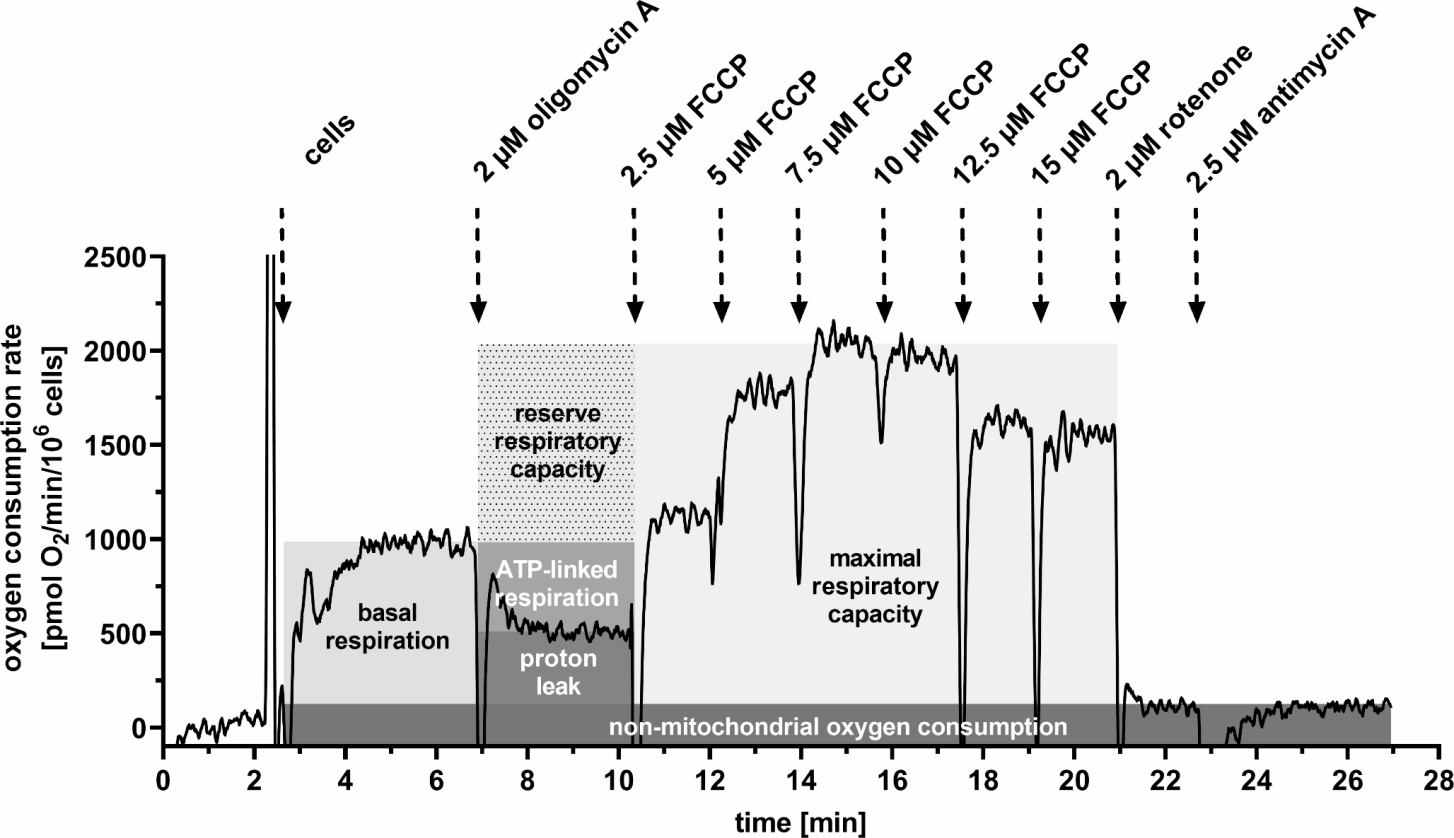
Protocol for measurement of oxygen consumption rate in intact cells. The figure depicts the representative measurement of oxygen consumption rate, along with explanation of respiration parameters that were derived from the recording. The arrows indicate the addition of cell suspension and of the specified concentration of the respiration modulators

**Fig. 3:**
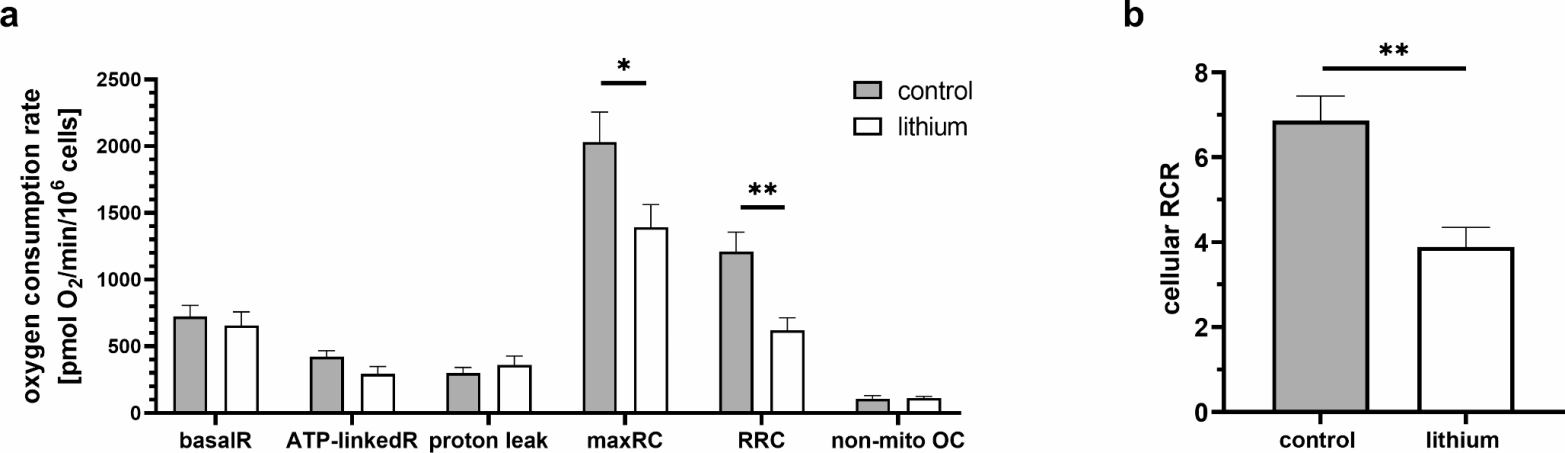
Rate of oxygen consumption in intact cardiomyoblast cells. (**a**) Absolute values of oxygen consumption rate per 10^6^cells: basal respiration, respiration linked to ATP production, proton leak, maximal respiratory capacity – maxRC, reserve respiratory capacity - RRC and non-mitochondrial oxygen consumption (**b**). The cellular respiratory control ratio (cellular RCR) was found to be significantly reduced in the cells exposed to 2 mM lithium in the culture medium. The values are represented by mean ± SEM from *N* = 8 values in each group. The statistical significance is indicated by: * 0.05>*P*>0.01; ** 0.01>*P*>0.001

Our next aim was to characterize the function of the cells by measuring the oxygen consumption. The rate of cellular respiration was measured in intact cells. We determined several parameters of respiration; their calculation is summarized in Fig. 2. A substantial impact was seen on the maximal respiration capacity (maxRC), which demonstrated a considerable decline in the presence of lithium (68.5% of control, unpaired t-test: *P* = 0.042; Fig. 3a). The major factor contributing to the alteration of maxRC was the reserve respiratory capacity (RRC). The control group exhibited a mean oxygen consumption rate 1210 ± 146 pmol O_2_ min^− 1^/10^6^ cells, whereas the lithium-treated group demonstrated a significantly reduced rate 620 ± 94 pmol O_2_ min^− 1^/10^6^ cells (*N* = 8, unpaired t-test: *P* = 0.0044; Fig. 3a).

The mitochondrial performance can be quantified by a parameter named cellular respiratory control ratio (RCR) (Hill et al. 2012). The RCR parameter was initially derived for isolated sarcosomes (Chance and Baltscheffsky 1958). It can also be calculated for the respiration of intact cells as a ratio of maxRC to proton leak (Brand and Nicholls 2011; Hill et al. 2012). A significant decline of cellular RCR was observed in the presence of lithium. Mean value for the control group was 6.86 ± 0.58, while the mean value for the lithium-treated group was 3.88 ± 0.46 (*N* = 8; unpaired t-test, *P* = 0.0013; Fig. 2b). These changes are related to the high lithium concentration, as they have not been observed under the therapeutic concentration of lithium (0.8 mM; Online Resource, SI3).

**Fig. 4:**
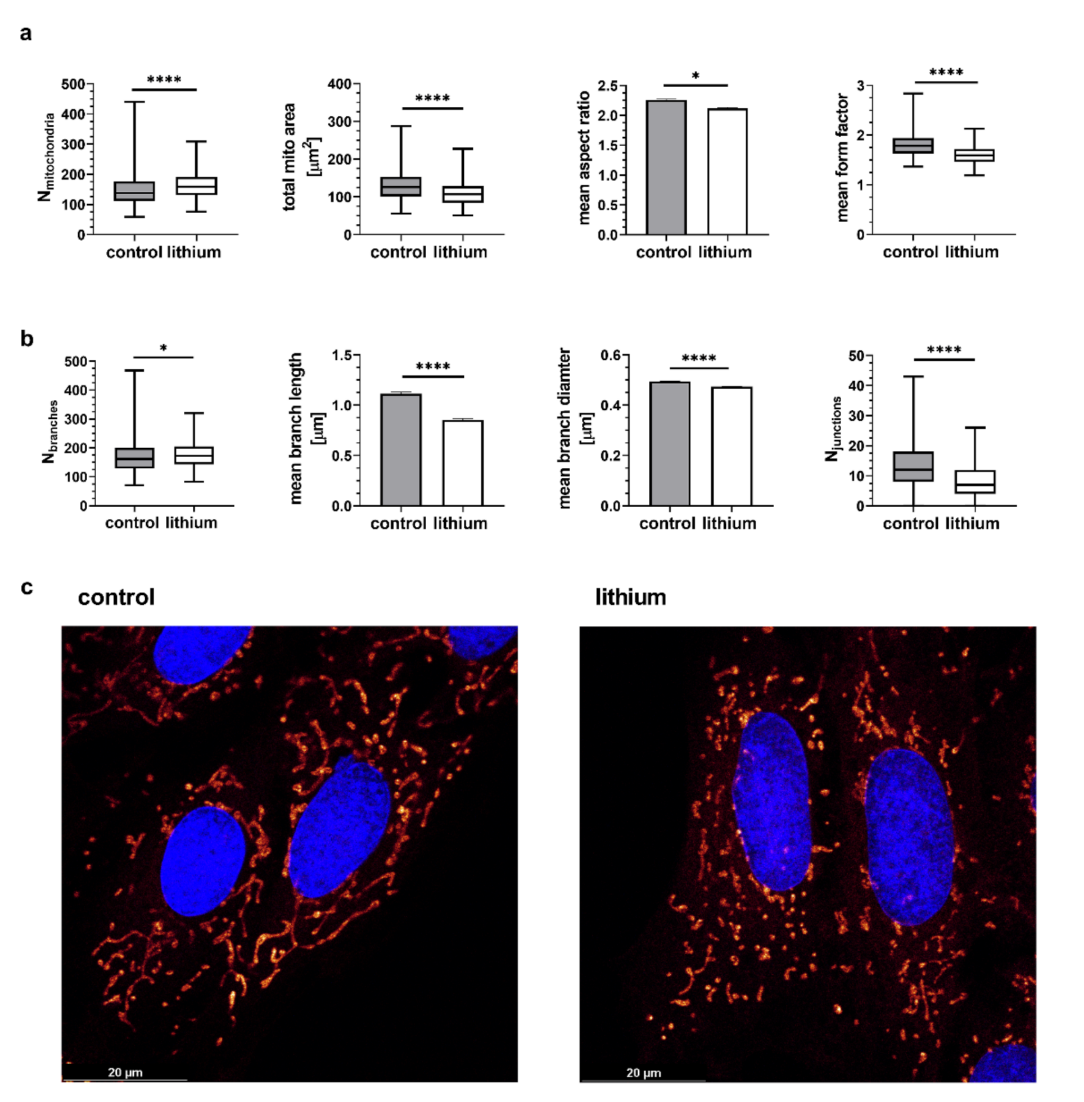
(**a**) Mitochondrial morphology parameters and parameters of cardiomyoblasts mitochondrial networks (**b**). Comparison of control and lithium-treated cardiomyoblasts was done on N_control_=255 and N_lithium_=250 cells. (**c**) Representative mitochondrial network of control cell and lithium-treated cell. The statistical significance is indicated by: * 0.05 > *P* > 0.01; **** *P* < 0.0001

An altered mitochondrial energetic state is frequently correlated with morphological changes. The mitochondrial networks were visualized using the fluorescent dye Mitotracker Deep Red. A summary of the morphological parameters is provided in Fig. 4. The calculations were performed on a total of *N* = 255 control cells and *N* = 250 lithium-treated cells. *Mitochondria analyzer* plugin offers two types of analysis – one that describes the mitochondrial morphology (Fig. 4a) and one that provides parameters of the mitochondrial network (Fig. 4b). Lithium-treated cells exhibited a greater number of mitochondria that were smaller in area (total mitochondrial area) and more rounded in shape (form factor, aspect ratio). Concerning the connectivity of the mitochondrial network, lithium treatment resulted in the shortening of branches that were less connected, thereby indicating that the mitochondrial network became fragmented. The representative mitochondrial networks are shown in Fig. 4c.

**Fig. 5:**
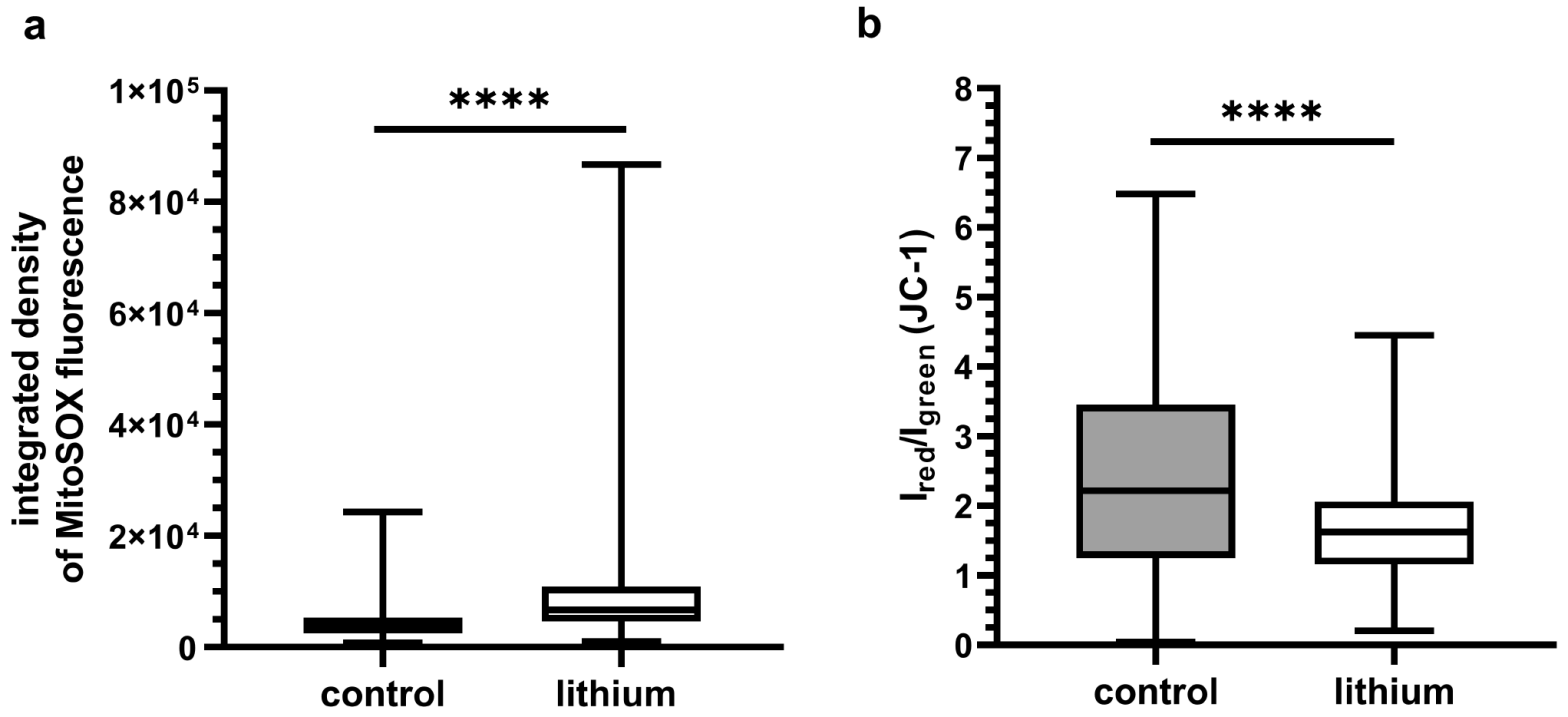
The level of superoxide radicals in mitochondria (**a**) and changes of mitochondrial membrane potential (**b**). Increased fluorescence of MitoSOX indicates an increase in superoxide radical level in the cardiomyoblast cells (N_control_=260; N_lithium_=224; t-test, **** *P* < 0.0001). JC-1 signal calculated as red/green fluorescence ratio was altered in the presence of lithium (N_control_ =325; N_lithium_=357; Mann-Whitney test, *P* < 0.0001)

A suboptimal bioenergetic state of a cell is frequently associated with an elevated level of reactive oxygen species (ROS). The production of superoxide radicals in mitochondria was markedly elevated in the presence of 2 mM LiCl in the culture medium, as compared to the control cells (Mann-Whitney test, *P* < 0.0001; Fig. 5a). Furthermore, the presence of lithium resulted in reduction in the JC-1 red/green fluorescence ratio, indicating less polarized mitochondria (Fig. 5b, Online Resource, SI4).

**Fig. 6:**
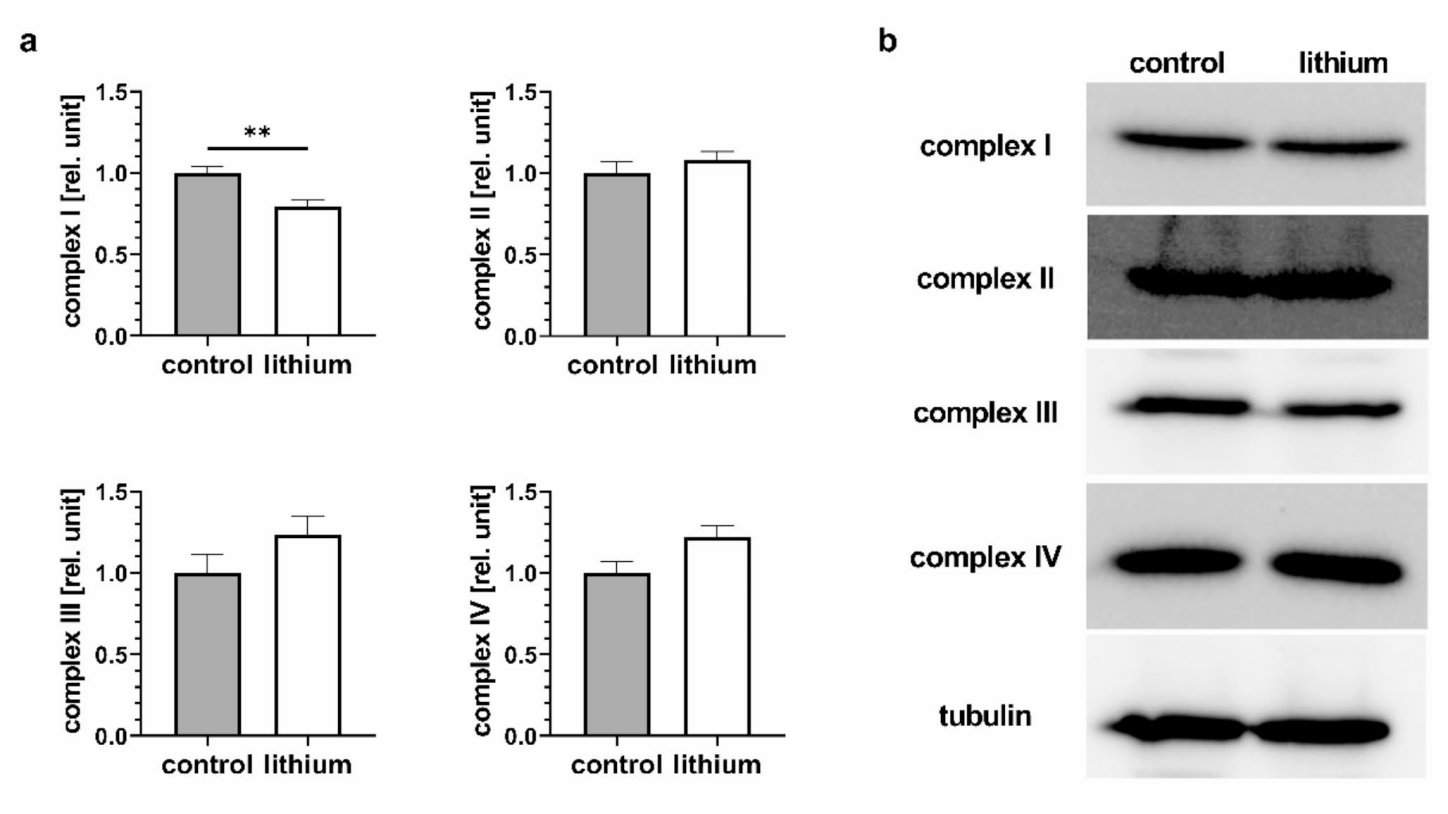
Activity (**a**) and protein level of ETC complexes (**b**). The effect of lithium on the activity of complexes I – IV of the electron transport chains, measured in lysates of isolated mitochondria (**a**). The activity is expressed as relative to control. A marked decrease of activity of complex I was detected (*N* = 4; t-test, *P* = 0.0097). For the other three complexes, there was no alteration (complex II: *N* = 5; t-test, *P* = 0.3878; complex III: *N* = 4; t-test, *P* = 0.1977; complex IV: *N* = 4; t-test, *P* = 0.0700). The statistical significance in graph is indicated by: ** 0.01 > *P* > 0.001. (**b**)

To elucidate the alteration in the mitochondrial parameters, we conducted the measurement of activity of individual electron transport chain (ETC) complexes (Fig. 6a). We found 20% decrease of complex I activity in lithium in comparison to control. The activity of complexes II, III and IV were not significantly altered, though complexes III and IV showed a trend of increased activity in lithium-treated cells; the protein level of individual complexes was unchanged (Fig. 6b, Online Resource, SI5).

**Fig. 7:**
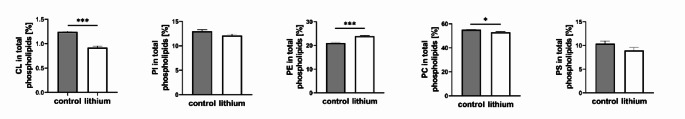
Changes in the relative phospholipid content. The relative proportion of different phospholipids in the overall cellular phospholipid content was altered in lithium-treated cells. The statistical significance is indicated by: * 0.05 > *p* > 0.01; ** 0.01 > *p* > 0.001; *** 0.001 > *p* > 0.0001. CL - cardiolipin, PC - phosphatidylcholine, PE - phosphatidylethanolamine, PI - phosphatidylinositol, PS - phosphatidylserine

It is established that the ETC complexes require optimal lipid composition to operate at their maximal possible rate (Horonyova et al. 2024; Senoo et al. 2020). We thus undertook an analysis of the lipid composition of control and lithium-treated cells. A decreased proportion of cardiolipin by 26% was observed in the overall phospholipid content (*N* = 5; unpaired t-test; *P* = 0.0002; Fig. 7, Online Resource, SI6), which is a lipid localized specifically to the mitochondrial membranes and is crucial for the formation and function of ETC supercomplexes (Houtkooper and Vaz 2008) and ATP synthase (Muhleip et al. 2019). Additionally, we observed a notable elevation in phosphatidylethanolamine (*N* = 5; unpaired t-test; *P* = 0.0005) and a reduction in phosphatidylcholine (*N* = 5; unpaired t-test; *P* = 0.0359; Fig. 7). The observed reduction in cardiolipin can be related to the alteration in ETC complexes activity.

## Discussion

The cardiotoxic adverse effects of lithium treatment in patients suffering from BD occur rarely. Such effects are typically associated with an elevated concentration of lithium in the blood serum. The level of lithium is standardly monitored on weekly basis at the beginning of treatment and at intervals of several months thereafter (Malhi et al. 2017; Tondo et al. 2019). At the cellular level, the BD is associated with mitochondrial malfunction and energetic dysregulation in neurons (Clay et al. 2011). It is, therefore, anticipated that the antipsychotic drugs exert an effect on mitochondria, as demonstrated in neuronal cells for many of these drugs (Bachmann et al. 2009; Inuwa et al. 2005; Li et al. 2007; Osete et al. 2021; Salimi et al. 2017; Wang 2007). The treatment of BD affects not only the brain but also other tissues, which can result in an emergence of adverse effects. The objective of this study was to examine the effect of higher than therapeutic concentration of lithium on mitochondrial function in cardiomyoblasts. These are more suitable for long-term experiments compared to more vulnerable isolated primary cardiomyocytes. Our objective was to examine the long-term effect (48 h) rather than an acute reaction. The best way to assess the mitochondrial functionality in the cells is to measure the oxygen consumption rate and quantify respiratory parameters (Brand and Nicholls 2011). The presented findings indicate that the exposure of cardiomyoblasts to higher than therapeutic dosage of lithium leads to changes in mitochondrial health, as evidenced by notable reduction in the RRC and a decline in the cellular RCR (Fig. 3). The changes in RRC have not been observed with lithium concentration within the therapeutic range (Online resource, SI3). RRC is correlated with the resilience of cells against stress (Marchetti et al. 2020; Nickens et al. 2013; Sansbury et al. 2011), its magnitude and regulation are dependent on the cell type (Hill et al. 2012; Pfleger et al. 2015). The optimal substrate or combination of substrates differs depending on the cell type. For instance, glucose, pyruvate or combination of both can result in varying magnitudes of RRC in neonatal rat cardiomyocytes, without significantly affecting basal respiration (Sansbury et al. 2011).

The functional alterations of mitochondrial respiration are frequently reflected in the morphology of the mitochondrial network (Benard et al. 2007; Xie et al. 2018). Our analysis revealed changes in the network morphology in the presence of 2 mM lithium (Fig. 4). The mitochondria in lithium-treated cells exhibited a reduction in size and a decrease in interconnectivity. The shortening of the mitochondria is regarded as an indication that the mitochondria are not functioning optimally (Fenton et al. 2021; Yapa et al. 2021). While studying the effect of lithium on the cardiomyocyte mitochondria, Salimi and colleagues concluded, based on a MTT test, that the activity of complex II was significantly inhibited by the presence of 1 mM lithium (Salimi et al. 2017). The MTT test has recently been revised (Ghasemi et al. 2021), resulting in the conclusion that it rather reflects the overall activity of the cellular/mitochondrial oxidoreductases and dehydrogenases. Here, we analyzed activities of four individual ETC complexes (Fig. 6). However, instead of reduced activity of complex II – succinate dehydrogenase, we observed decrease in activity of another mitochondrial enzyme, complex I – NADH dehydrogenase. Therefore, it is more likely that the reduced activity of complex I contributed to the observed decrease in formazan absorbance (Fig. 1a).

The overproduction of superoxide radicals may also be the result of the improper function of complex I, a primary source of superoxide radicals in mitochondria (Nolfi-Donegan et al. 2020). The level of superoxide radicals in lithium-treated cardiomyoblasts (Fig. 5a) confirmed the reported increase in ROS measured in isolated cardiac mitochondria from rat caused by acute exposure to less than 1 mM lithium (Salimi et al. 2017). In addition, malfunction of complex I can be the cause of reduced mitochondrial membrane potential (Fig. 5b).

The efficiency of the ETC depends on the lipid composition of mitochondrial membranes (Mileykovskaya and Dowhan 2014) and it has been reported that lithium can influence lipid metabolism (Brown and Tracy 2013; Vosahlikova et al. 2021). In our study, treatment with 2 mM lithium induced changes in phospholipid composition, with the most prominent effect being a significant decrease in mitochondrial lipid cardiolipin (Fig. 7). Cardiolipin is essential for the optimal functioning of the ETC supercomplexes or Krebs cycle (Kanovicova et al. 2022; Mileykovskaya and Dowhan 2014; Raja et al., 2017; Senoo et al. 2020). A reduction in cardiolipin level was observed to result in a decline in RRC in cerebral microvascular endothelial cells (Nguyen et al. 2016). Low content of cardiolipin in the mitochondrial membrane may result in a suboptimal arrangement of supercomplexes. These can function less effectively under conditions of maximal respiration, even though they function well at the basal rate. The observed reduction in the proportion of cardiolipin (Fig. 7) may be, apart from complex I activity, also a contributing factor to the lower rate of maximal respiration and RRC that was observed (Fig. 3).

The alterations of mitochondrial network morphology, the elevation of superoxide radicals within mitochondria, less polarized mitochondria, reduced activity of complex I and a decline in RRC collectively suggest that the cells have impaired metabolism and may be susceptible to failure in face of stress or when confronted with higher energetic demand (e.g. during physical activity). As was claimed in (Akkouh et al. 2020; Brown and Tracy 2013; Shalbuyeva et al. 2007), lithium has a broad range of actions. Thus, its cardiotoxic effect is likely a result of parallel, yet different processes. The mechanism by which higher lithium dosage induces the changes in respiration and mitochondrial morphology is connected with the complex I activity as well as altered phospholipid composition.

## Conclusion

Lithium affected mitochondrial bioenergetics without significantly altering the basal rate of respiration. The morphology of the mitochondrial network exhibited fragmentation of mitochondria. The observed decline in the reserve respiratory capacity, reduced mitochondrial polarization and increased superoxide radical production point to the malfunction of mitochondrial energetics. Exposure to lithium was associated with the reduction of cardiolipin level, which is crucial for the optimal function of respiratory chain supercomplexes. The decreased reserve respiratory capacity renders the cells vulnerable to higher energetic demands, which may contribute to the cardiotoxic effects of the lithium treatment.

## Electronic supplementary material

Below is the link to the electronic supplementary material.

Supplementary Material 1

## Data Availability

Data are available upon request on email adress: zuzana.tomaskova@savba.sk.

## Abbreviations

BD: Bipolar disorder
DMEM: Dulbecco modified Eagle medium
ETC: Electron transport chain
FCCP: Carbonyl cyanide 4–(trifluoromethoxy) phenylhydrazone
MTS: 3–(4,5–dimethylthiazol − 2–yl)–5–(3–carboxymethoxyphenyl)2–(4–sulfophenyl)–2 H–tetrazolium
MTT: 3–(4,5–dimethylthiazol–2–yl)–2,5–diphenyltetrazolium bromide
PBS: Phosphate buffered saline
RCR: Respiratory control ratio
ROS: Reactive oxygen species
RRC: Reserve respiratory capacity
SDH: Succinate dehydrogenase
