# Supplementary material for: Lithium compromises the bioenergetic reserve of cardiomyoblasts mitochondria
Source: J Bioenerg Biomembr. 2025 Jan 24;57(1):27–38. doi: 10.1007/s10863-024-10050-x (PMC11829843; doi:10.1007/s10863-024-10050-x)
Supplement: Supplementary file 1 — Supplementary Material 1 [file 10863_2024_10050_MOESM1_ESM.pdf]

Lithium compromises the bioenergetic reserve of cardiomyoblasts mitochondria

Supplementary information

Journal of Bioenergetics and Biomembranes

Grman Marian, Balazova Maria, Horvath Anton, Polcicova Katarina, Ondacova Katarina, Stepanovsky Jakub, Sevcikova Tomaskova Zuzana

Affiliation of corresponding author Zuzana Sevcikova Tomaskova:

Institute of Molecular Physiology and Genetics, Centre of Biosciences of the Slovak Academy of Sciences, Dubravska cesta 9, 840 05 Bratislava, Slovakia

Email: zuzana.tomaskova@savba.sk

SI1. Lithium-induced concentration dependent changes of cell count and cell proliferation after 48 h of exposition.

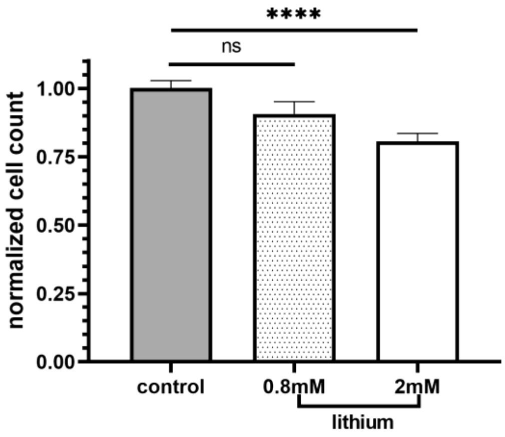

The number of cells after 48 h exposition to lithium represented 90 ± 4.5 % of control in 0.8 mM LiCl (Sidak's multiple comparison test following one-way ANOVA; P=0.1051; $N_{control}$=21; $N_{0.8Li}$=12) and 80 ± 3 % of control in 2 mM LiCl (Sidak's multiple comparison test following one-way ANOVA; P<0.0001; $N_{control}$=21; N_{2Li}=17).

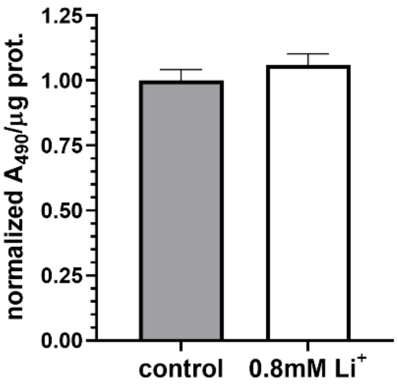

Cell proliferation assay showed no difference between control and 0.8 mM lithium-treated group (t-test; P=3396; N=7). The data were normalized according to (Valcu & Valcu, 2011).

SI2. Detection of dead cell proportion by staining with propidium iodide (red) with Hoechst (blue)

Comparison of control cells and cells treated with 2 mM LiCl. Representative images, overlay of red and blue channels. Nuclei stained also with both Hoechst and propidium iodide appear in lilac color.

control

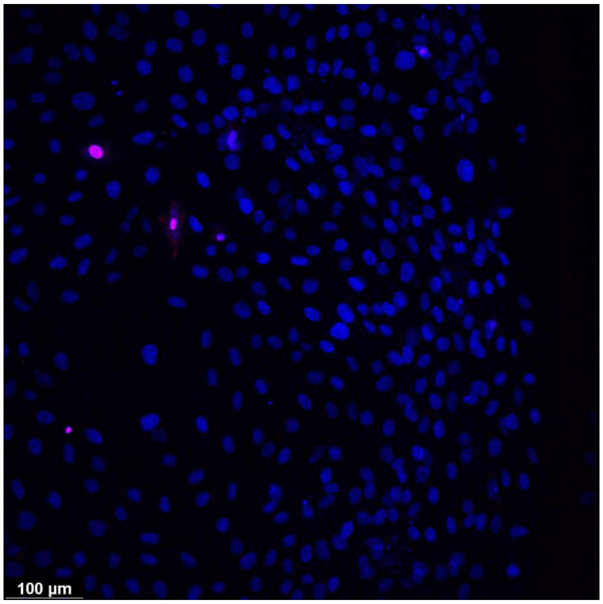

2 mM lithium

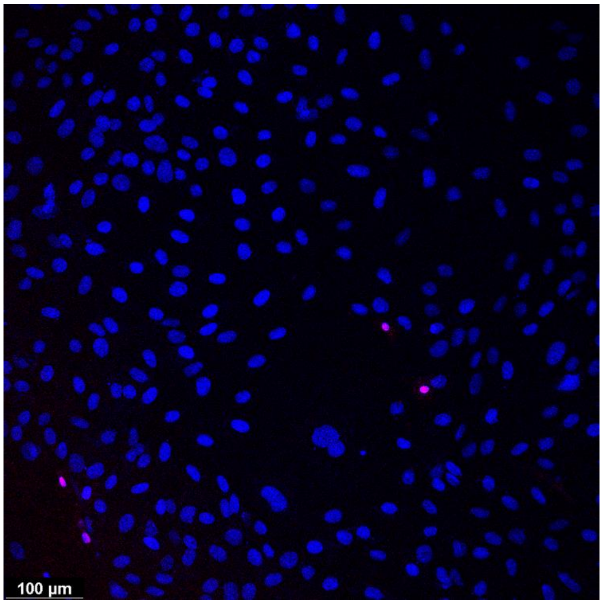

SI3. Effect of 0.8 mM LiCl on oxygen consumption rate in intact cells.
The respiration of intact cells was measured as described in the *Material and Methods* section, but lithium-treated group was exposed to therapeutic concentration of lithium, 0.8 mM instead of 2 mM lithium. No significance was detected between the control and lithium-treated group (N=3, t-tests; basal R: P=0.1236; ATP-linked R: P=0.0785; proton leak: P=0.5041; maxRC: P=0.9064; RRC: P=0.7176; non-mito R: P=05978). In graph **(a)** are the different respiration parameters, in graph **(b)** is comparison of cellular respiratory control ratio (t-test; P=0.3144; N=3).

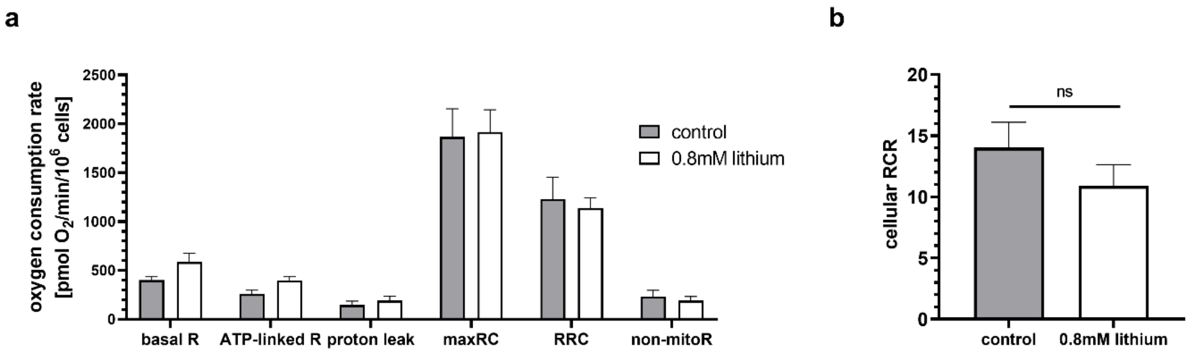

SI4. JC-1 staining and changes of intensity ratio induced by addition of FCCP.

FCCP is a protonophore which dissipates the mitochondrial membrane potential. The red fluorescence of corresponds to aggregates formed by JC-1 in polarized mitochondria. Green fluorescence of JC-1 originates from the monomers of this dye. Representative images of cardiomyoblast cells loaded with JC-1 – the addition of up to 15 μM FCCP caused decrease of JC-1 aggregate fluorescence (red).

control

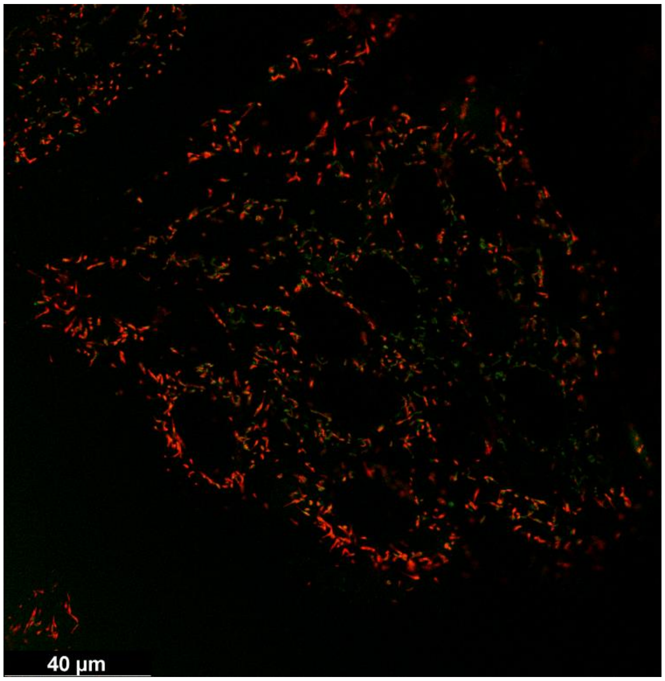

2 mM lithium

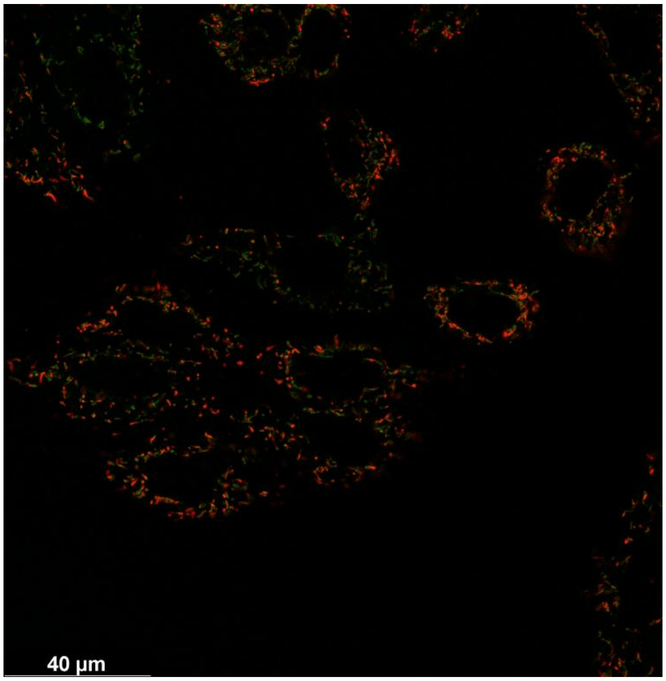

15 μM FCCP, control

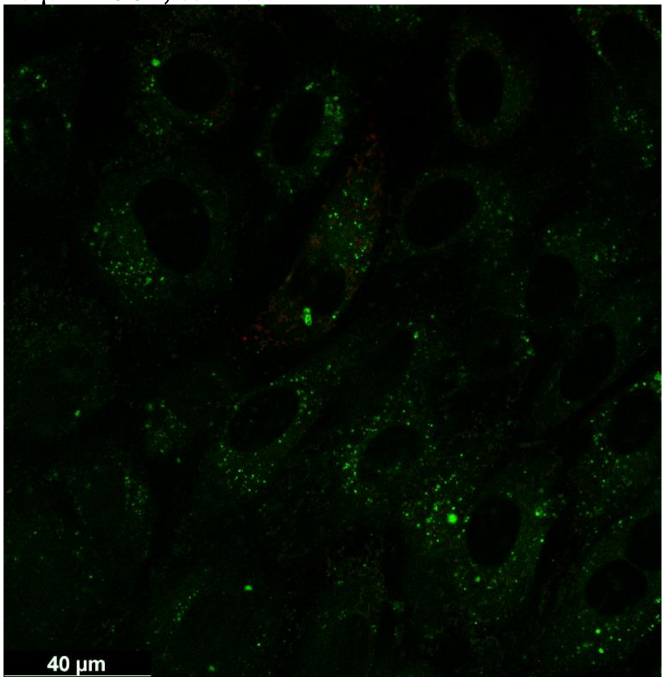

15 μM FCCP, 2 mM lithium

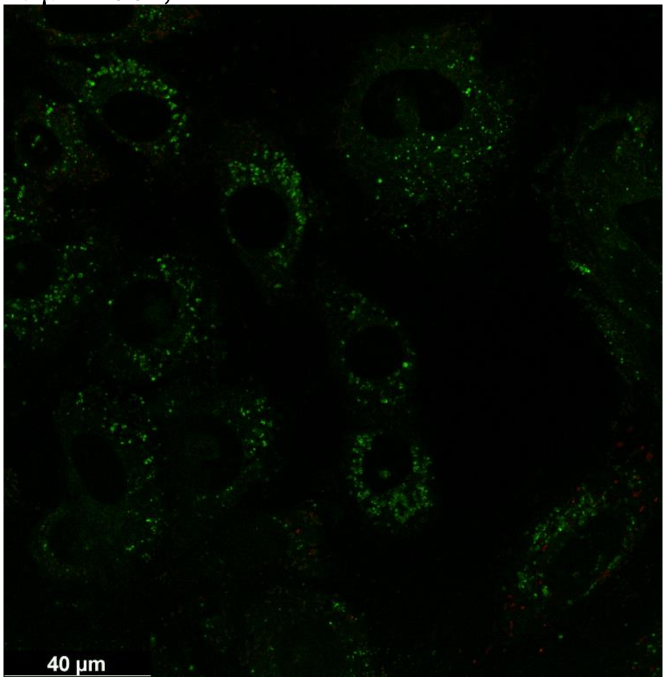

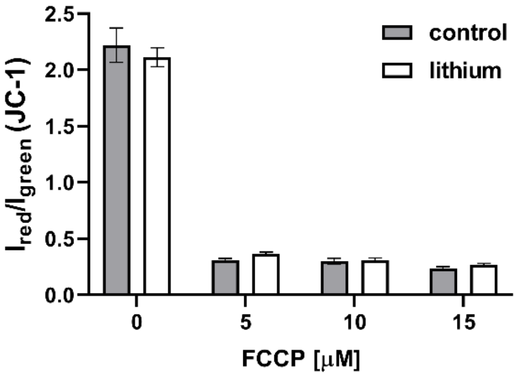

The ratio of red/green fluorescence intensity deceased in presence of FCCP. The data are obtained from 44 – 77 cells, depending in the group).

SI 5. Quantification of protein level of ETC complexes.

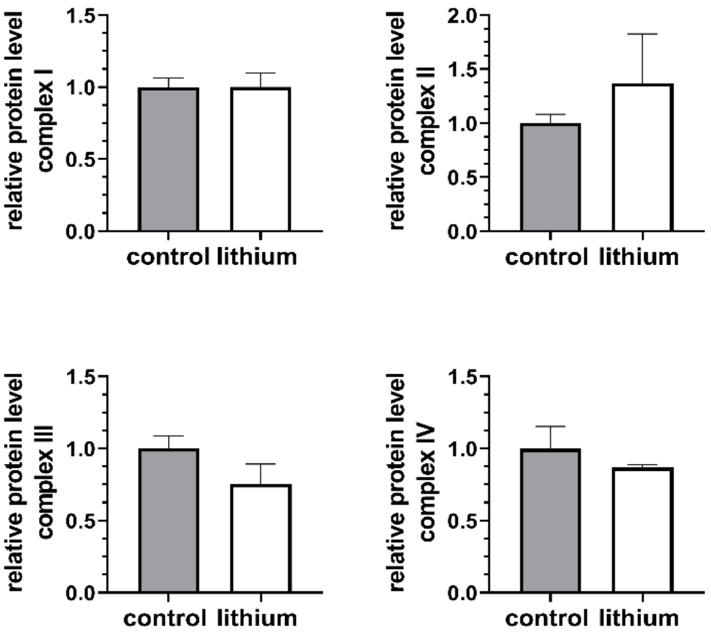

No alterations were detected protein levels of ETC complexes determined by western blot analysis. The signal from blot images was first normalized to tubulin level and then compared to control.

Complex I:
N=6; t-test, P=0.9994
Complex II:
N=3, t-test, P=0.4665
Complex III:
N=3; t-test, P=0.2310
Complex IV:
N=3; t-test; P=0.4394

SI 6. Thin layer chromatograms.

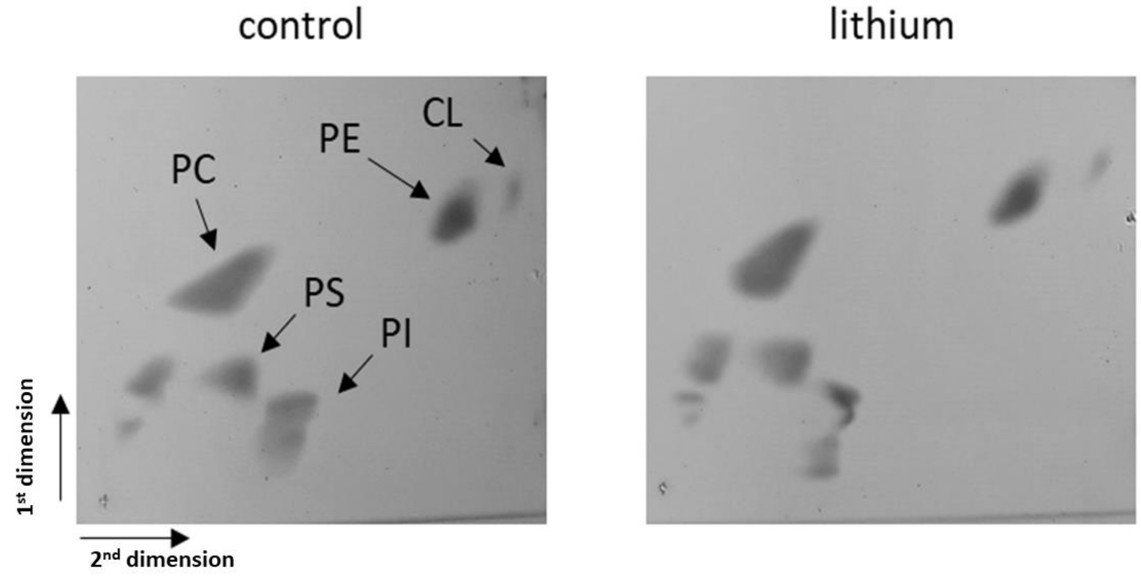

The separation of different phospholipids and their relative content was analyzed by thin layer chromatography. The effect of 2 mM lithium on the composition of cellular phospholipids is demonstrated on the representative chromatograms. Arrows 1. and 2. indicate the sequential directions of separation.
